# Acceptability of Internet-Based Therapy: Considerations Expressed by Individuals Struggling with Gambling

**DOI:** 10.1177/14550725261465112

**Published:** 2026-08-10

**Authors:** André Syvertsen, Bjørn Solberg-Hansen Fondenes, Ståle Pallesen

**Affiliations:** 1 Department of Psychosocial Science, 1658 University of Bergen, Bergen, Norway; 2 Norwegian Competence Center for Gambling and Gaming Research, 1658 University of Bergen, Bergen, Norway; 3 Department of Addiction Medicine, 60498 Haukeland University Hospital, Bergen, Norway

**Keywords:** gambling disorder, treatment attitude, ehealth, online therapy, qualitative survey

## Abstract

**Aims:**

Internet-based therapy (IBT) is effective in reducing symptoms of gambling disorder (GD), yet limited acceptability may constrain treatment uptake and engagement. This study explored perspectives on the expected acceptability (defined as perceiving the intervention as appropriate) of IBT for GD.

**Methods:**

Twenty-eight individuals with current or lifetime gambling problems responded to open-ended questions in a qualitative survey, including reflections on a vignette describing a hypothetical patient receiving IBT for GD.

**Results:**

Data were analyzed using thematic analysis and we identified three themes. The theme “Urgent matters” reflected participants’ desire for more emphasis on crisis management and harm reduction early in IBT. “Keeping the same standard” indicated that participants’ content preferences match what is typically offered in cognitive behavioral therapy, but with concerns that online delivery might compromise treatment quality. “Risk of pseudotherapy” reflected perceptions that IBT is more susceptible to falsely appearing effective compared to face-to-face therapy. Participants conveyed an understanding that GD characteristics of avoidance and motivational ambivalence can undermine IBT engagement, particularly in the absence of social contact with a therapist or others with lived experience.

**Conclusions:**

The findings suggest that assessments of IBT acceptability for GD and potential interventions aiming to increase acceptability should account for gambling-specific challenges.

Internet-based therapy (IBT) is available for multiple psychiatric conditions, including gambling disorder (GD) where it shows comparable effectiveness as traditional face-to-face therapy when guided by a therapist (Sagoe et al., 2021). IBT for GD offers unique benefits such as greater geographical coverage for treatment, quality assured standardized treatment content, and time and place flexible treatment delivery compared to face-to-face therapy (Van Der Maas et al., 2019). These benefits may increase treatment uptake, which has been estimated to be as low as one in 25 among individuals with moderate risk gambling and one in five among individuals experiencing gambling problems (Bijker et al., 2022). IBT uptake appears to be dependent on potential users viewing it as acceptable, which, in this context, refers to providers and recipients of health-related interventions perceiving an intervention to be appropriate for their condition or goals (Sekhon et al., 2017). Such perceptions are based on either expected or experienced emotional and cognitive responses to the intervention. Mapping expected acceptability is important because this can inform amendments to how internet-based treatment is communicated to potential users and how it is designed

## The Modern Landscape of Internet-Based Treatment

IBT are interventions delivered online that leverage technological solutions for greater interactivity for patients and clinicians (Ebert et al., 2018). Many IBTs for psychological disorders are grounded in cognitive-behavioral therapy (CBT) and include some form of therapist guidance (Ebert et al., 2018). The latter is usually provided once a week and is asynchronous, meaning that messages are not exchanged in real time. The primary goal of therapist guidance has generally been to boost adherence to the treatment program. In practice, clinicians will clarify issues, provide feedback on completed work, encourage the patient, and ensure continued progress. Communication in guided IBT can occur via text messaging, in-program chat, telephone or video calls. In some cases, IBT is fully automated (completely unguided). IBT may be delivered as a standalone intervention, combined with face-to-face therapy (blended care), or integrated into a stepped-care sequence (Ebert et al., 2018). Although few studies directly compare different configurations of these features, therapist guidance and the amount of time spent providing it appears to be positively associated with treatment effects (Ebert et al., 2018; Sagoe et al., 2021). IBT is probably a poor fit for individuals with limited digital literacy (Nilsson et al., 2023), and further research is needed regarding its limitations and potential adverse effects. Common concerns have included inadequate assessment and response to suicidality, the risk that non-response may foster negative attitudes toward therapy, treatment-related harm, reduced self-efficacy due to reliance on digital tools, and insufficient data protection. Still, current evidence suggests that IBT is not associated with a higher risk of such adverse outcomes than face-to-face therapy (Ebert et al., 2018).

IBT has its roots in bibliotherapy, where physical written materials and workbooks were first made available online before being revised or replaced by uniquely digital solutions (Andersson et al., 2019). This development has unfolded in tandem with a broader expansion of IBT offerings, a trajectory further accelerated by the COVID-19 pandemic (Smith et al., 2022). The pandemic reduced access to traditional face-to-face treatment for gambling disorder due to lockdowns and social distancing measures. This prompted wider adoption of telehealth solutions, including IBT, with lasting effects on the field (Smith et al., 2022). Technical solutions have now advanced considerably, leveraging unique capabilities of modern web applications and smartphones. IBT can now incorporate a range of tools, such as digital reminders, standardized feedback, and interventions tailored to time of day, user location or individual risk factors (McCurdy et al., 2023; McDonald et al., 2020). Still, IBTs grounded in CBT remain the most robustly evaluated format for gambling disorder (Sagoe et al., 2021). Recent evaluations of IBTs based on CBT for gambling disorder confirm treatment efficacy while utilizing higher sample sizes, routine care settings, active comparisons, and/or long-term follow-up and comparisons with registry data (Korvuo et al., 2023; Mide et al., 2023; Molander et al., 2024).

## The Expected-Experienced Acceptability gap

The theoretical framework of acceptability (TFA) elaborates on the multi-faceted nature of acceptability (Sekhon et al., 2017). TFA comprises seven components that subsume whether an intervention is viewed appropriate for one's goals: Affective attitude (feelings about the intervention), burden (participation effort), ethicality (value system fit), intervention coherence (intervention understanding), opportunity costs (drawbacks), perceived effectiveness, and self-efficacy (confidence in participation). A key feature within TFA is that acceptability can be assessed prospectively (expected acceptability prior to engagement) and retrospectively (experienced acceptability after participation). There appears to be a divide between expected and experienced acceptability: experience with IBT is associated with favorable attitudes towards it, whereas lack of experience is associated with more negative or ambivalent attitudes towards IBT.

Experienced acceptability has generally been found to be favorable for IBTs for GD, including guided and unguided formats, traditional module-based CBT programs, and newer smartphone apps with time-tailored features (Rodda et al., 2025; Rolvien et al., 2024; Stenbro et al., 2023).

Expected acceptability of IBT in the general population has remained low over time, despite the concurrent technological advancement and expansion of IBT (De Jesús-Romero et al., 2022; Lincke et al., 2022; Molloy & Anderson, 2022; Steubl et al., 2025). Relatedly, repeated measurements have shown comparable expected acceptability of IBT before and after the COVID-19 pandemic (Molloy & Anderson, 2022). Individuals that report negative expected acceptability emphasize lack of perceived effectiveness and opportunity costs to independence, privacy/confidentiality, and human contact (De Jesús-Romero et al., 2022; Starvaggi & Lorenzo-Luaces, 2025; Steubl et al., 2025). Although privacy/confidentiality and self-reliant recovery are commonly emphasized strengths to IBTs (Van Der Maas et al., 2019), these factors can also constitute barriers when the individual has concerns about data privacy or about receiving help in general (Borghouts et al., 2021). Among broader correlates, higher age and lower education have been associated with lower expected acceptability of IBT, which might reflect barriers related to digital literacy (Lincke et al., 2022; Steubl et al., 2025). The expected–experienced acceptability gap suggests that preconceived views or expectations may hinder treatment uptake, underscoring the value of understanding how people that struggle with gambling but without actual IBT treatment experience evaluate IBT.

## Expected Acceptability of Internet-Based Treatment for Gambling Problems

People gambling or struggling with gambling are likely to hold unique views and challenges that are poorly captured by current acceptability studies using general-population samples or samples experiencing other types of mental health difficulties. Shame and stigma are particularly strong barriers for treatment seeking in gambling and one population study indicates that those perceiving greater stigma report more openness to IBT than those perceiving less stigma (Klein & Cook, 2010; Loy et al., 2018). This is consistent with the expectation that greater anonymity can lower the barrier for treatment. At the same time, because gambling is increasingly conducted online (Pallesen et al., 2021), some individuals struggling with gambling may worry that engaging with therapy on the internet could expose them to gambling cues or environments that facilitate relapse (Sanchez et al., 2019). Other barriers can include a wish to handle the problem by oneself, unwillingness to admit problems, concern about what goes on in treatment or its quality/efficacy, difficulty in sharing problems or talking about personal issues, lack of knowledge about treatment options, and/or practical issues around attending treatment (Suurvali et al., 2009).

There are few studies investigating expected acceptability of IBT among individuals struggling with gambling. Existing studies indicate that those struggling with gambling report higher expected acceptability of IBT compared to the general population, although a considerable proportion remain skeptical (Hawker et al., 2025a; Sanchez et al., 2019). Positive expected acceptability is reflected in perceived benefits to IBT, including IBT offering greater understanding of one's own gambling problem and anonymity making it easier to share feelings (perceived effectiveness), IBT offering greater treatment availability, time- and context-independent support, and reduced practical costs (lower opportunity costs), and IBT offering effective professional guidance for self-help (lower burden and supporting self-efficacy) (Hawker et al., 2025a; Sanchez et al., 2019). Conversely, negative expected acceptability is reflected in views that IBT is less professional than traditional therapy (perceived effectiveness) and that unguided formats create difficulties understanding therapeutic terms and concepts (intervention coherence). Beyond aggregate acceptability levels, the salience of specific acceptability components may also vary as a function of individual differences: Symptom severity of gambling problems is positively associated with perceiving IBT benefits to anonymity (less opportunity costs), whereas higher gambling expenditure and gambling related harms is positively associated with perceiving IBT as inferior to face-to-face treatment in promoting long-term change (lower perceived effectiveness), perceiving IBT as conducive to isolation/loneliness (more opportunity costs), and perceiving IBT as causing difficulties in translating treatment skills to daily life (lower self-efficacy) (Hawker et al., 2025a).

Those struggling with gambling have suggested that IBT acceptance can be improved (i.e., they would be more inclined to engage) if the program was easy to learn, confidentiality was ensured, and that they could control the amount of personal information shared. They have additionally suggested broad elements for program design to this effect: 24/7 availability, therapist guidance, options for real-time communication, exercises and interactive tasks, resources for concerned significant others, and support for comorbid conditions (e.g., anxiety, depression) (Sanchez et al., 2019).

## The Norwegian and Nordic Context

The present study was conducted in Norway where help-offerings for gambling problems exist through low-threshold pathways, self-help organizations, and within specialist healthcare. Individuals struggling with gambling can receive financial guidance from the Norwegian Labor and Welfare Administration (nav.no) (e.g., in terms of debt relief application). At the time of the present study, low-threshold pathways notably included a national helpline and chat service offering guidance for individuals struggling with gambling and concerned significant others (www.hjelpelinjen.no). Self-help groups for gambling problems are offered digitally and physically through Gamblers Anonymous and Gambling Addiction Norway, with the latter representing the most widely available self-help group and that follow an atheoretical model (Syvertsen et al., 2020). CBT and motivational interviewing are the most common treatment approaches within specialist healthcare for GD in Norway and treatment access is relatively good with 79% of publicly listed outpatient and inpatient facilities in Norway confirming such offerings in 2025 (Torvund et al., 2018; Tørdal & Pallesen, 2025). IBT offerings are less prevalent, although Blue Cross Norway has offered a hybrid treatment nationally with phone consultations and online material since 2021 (spillbehandling.no). Within the Nordic context, IBT for gambling disorder appears well-developed in Sweden where several randomized controlled trials have been reported since 2008 (Sagoe et al., 2021). Overall, IBT for gambling disorder appears to be growing in the Nordic countries, as Denmark (Stenbro et al., 2023), Sweden (Molander et al., 2024), and Finland (Mide et al., 2023) report recent evaluations of IBT programs. We are unaware of any IBT for gambling disorder offerings in Iceland. The present study was conducted when the development of a novel and fully internet-based and therapist guided CBT program was starting in Norway, aiming to expand such IBT access in Norway.

The aim of the present study was to inform the development of the new IBT program for GD in Norway, at the same tome as addressing the lack of qualitative studies on expected acceptability for IBT for GD internationally. The study was guided by the following research question for capturing expected acceptability: how do individuals with current or previous gambling problems evaluate internet-based therapy as appropriate for their condition and goals, including what factors do they identify as facilitating or hindering its acceptability? Our focus is on preferred content, structure, and anticipated benefits and challenges in IBT for GD. This focus enabled the study to elucidate multiple components of the multi-faceted acceptability construct (Sekhon et al., 2017).

## Methods

### Study Procedure and Design

The present study was based on a qualitative survey administered February 2023 and hosted on SurveyXact (surveyxact.com). The study employed purposive sampling of individuals 18 years or older with current or lifetime gambling problems among members of Norwegian self-help groups for gambling problems, organized by the non-governmental organization Gambling Addiction Norway (Patton, 2002). Recruitment was conducted with the assistance of administrative personnel at Gambling Addiction Norway who disseminated a flyer with study information through internal online channels and physical group meetings (for more information on Gambling Addiction Norway) (Syvertsen et al., 2020). The flyer contained a link to the online survey which also included an informed consent sheet with more detailed study information. Potential participants were informed that the survey was anonymous specifically targeting individuals with lived experience concerning gambling problems and that the aim of the study was to assess such individuals’ perspectives on development and content of IBT for gambling disorder. They were informed that previous experience with IBT was not a requirement to participate. Participants were compensated with a universal gift card valued 250 Norwegian kroner upon survey completion. Twenty-three participants completed the survey. An additional five answered two to five open-ended questions but did not provide background information. In total, 28 participants contributed open-ended responses in the study. The current study was exempted from ethical approval in accordance with the Norwegian Centre for Research Data's guidelines for anonymous surveys. Participants provided informed consent.

The study took a phenomenological approach emphasizing participants’ perspectives and meaning-making (Creswell & Poth, 2016). This consists of describing common experiences among participants relating to a specific phenomenon, here: Expectations and considerations that the participants foresee when engaging in IBT for GD. This includes “what” they consider IBT is and should be, and “how” they expect a person struggling with gambling would experience it. Participants were not asked to share their own experiences with therapy for GD, but it was assumed that their lived experience struggling with gambling and help-seeking would inform their answers about the content, structure, benefits, and drawbacks of IBT for GD. The study questions were designed to prompt participants’ perspectives on IBT as it relates to GD characteristics especially (see “Open-ended questions on IBT for GD and vignette”). The phenomenological approach enables us to draw on the participants’ perspectives, without imposing strict definitions on IBT that could constrain their perspectives.

### Measures

#### Demographic Information

Demographic measures included age (slider ranging 15–100 years), gender (“man,” “woman,” “other”), and ethnicity. The latter was assessed with the question “Where were you born?” with options: “Norway,” “Nordic country, outside Norway,” “European country, outside Nordic countries,” “Africa,” “Asia,” “North America,” “South- or Middle America” and “Oceania.”

#### Help-Seeking Experience

Participants were asked if they had previously participated in IBT for any condition (“yes,” “no”), if they had participated in face-to-face therapy (“yes,” “no”), and how many self-help groups they had attended (slider ranging from 1–100, with an option to state “above 100 meetings”).

#### Gambling Problems

Gambling problems was assessed with the Problem Gambling Severity Index (PGSI) (Ferris & Wynne, 2001). Participants were first asked “Have you ever experienced problems with gambling?” with options: (1) “yes, within last 12 months,” (2) “yes, more than 12 months ago,” and (3) “no.” Participants responding (1) or (2) were then asked to consider their relevant timeframe and respond to the nine PGSI items assessing problematic gambling behavior and negative consequences from gambling, in which items are scored on a four-point scale ranging from (0) “never” to (3) “always.” PGSI scores ≥ 8 constituted the categorical distinction of having gambling problems.

#### Open-Ended Questions on IBT for GD and Vignette

The survey included five open-ended questions assessing participants’ perspectives on IBT for GD, two questions related to a vignette about a hypothetical patient seeking help for GD through IBT (see below), and one optional question covering any additional perspectives that the participant might have on IBT for GD. The eight questions concerned what participants thought would be important topics to include in therapy, how the content should be structured, what type of support they think therapists should provide in therapy, if the therapy program should address issues beyond GD itself, and the potential benefits and drawbacks to offering a program without therapist support (i.e., pure self-help). Translated questions and a vignette are provided in the Supplementary material (Doc. S1).

The vignette details the story of “Petter” who develops a gambling problem throughout his adolescence and early adulthood while transitioning from electronic gaming machines to online gambling. “Petter” seeks therapy after being pressured by his wife, and experiences ambivalence towards gambling and therapy. The vignette was intended to facilitate how participants make meaning out of IBT in relation to GD specifically. Vignette development was informed by the clinical experience of AS and BF when working with GD in the Norwegian setting, as well as empirical findings on developmental pathways for GD, motivation and readiness for change, and factors influencing treatment-seeking (Blaszczynski & Nower, 2002; Loy et al., 2018; Nower et al., 2022; Petry, 2005). Participants were asked to rate how realistic they found the vignette using a slider ranging from 1 (“very unrealistic”) to 10 (“very realistic”). Participants perceived the vignette as highly realistic (*M* = 8.8 (*SD* = 1.8)).

### Data Analysis

Data were analyzed with thematic analysis in line with the step-by-step description of thematic analysis by Braun and Clarke (2021): (1) familiarization with the data (reading and making initial notes on participants’ responses); (2) coding (systematically identifying ideas that provide meaningful descriptions of the data); (3) generating initial themes in the material; (4) critically re-evaluating themes (going back to the material to verify that codes and themes correspond and make sense in the material); (5) refining, defining and naming themes; and (6) writing the report. An inductive approach was used, implying that data content formed the basis for code- and theme development rather than existing theory. Coding involved categorizing all statements relevant to the research question focusing on expressed (semantic) rather than underlying (latent) content, two example codes were “Online therapy is only for highly motivated” and “Recovery takes time.” Codes were grouped into themes by shared underlying meaning. For example, the aforementioned codes were grouped in the theme “Risk of pseudotherapy” because these codes and others reflected an idea that IBT risks superficially addressing GD characteristics such as ambivalence and relapse. We identified themes according to a central organizing concept, which reflects an idea that can unify patterns in the data that coincide or even diverge (Braun & Clarke, 2021). Data analysis was conducted by AS and using NVivo, version 14 (https://lumivero.com/products/nvivo), as the qualitative data analysis software for organizing codes and themes. The analytical proceedings were guided by the sense-making and significance of participants’ statements in relation to the research question over the frequency of the statements or word count in answers. Still, we also report frequencies for transparency in the results. This has been systematized in the reporting so that “a few” corresponds to 1–4, “several” corresponds to 5–10, “many” corresponds to 11–15, and “most” corresponds to 16 or above. Participant answers to each open-ended question ranged from one word to 176 words, with the total word count across the data material being 6487 words.

## Results

The study sample characteristics are provided in Table 1 for the 23 participants reporting background characteristics. The results provided in Table 1 indicate that most participants were men (65%), had life-time gambling problems (70%), reported severe problem intensity (median GDSI score 21 of 27 maximum), were born in Norway (87%), had attended many self-help meetings (median 50 meetings), and had prior experience with face-to-face therapy for GD (87%).

**Table 1.** Sample Characteristics.

Sample	*n* = 23
Median age (years) [IQR]	45 [39, 56]
Gender	
Man	15 (65%)
Woman	8 (35%)
Ethnicity^1^ (% of sample)	
Norway	20 (87%)
Nordic country, outside Norway	2 (9%)
Asia	1 (4%)
Experience with IBT	4 (17%)
Experience with face-to-face therapy	20 (87%)
Median self-help meetings attended [IQR]^2^	50 [25, 98]
Gambling problems timeframe	
Current (last 12 months)	6 (26%)
Lifetime (over 12 months ago)	16 (70%)
No problem	1 (4%)
Median GDSI sum score [IQR]^3^	21 [18, 23]

*Note*. IQR: interquartile range; PGSI: Problem Gambling Severity Index;

1 Participants could also respond “European country outside Nordic countries,” “Africa,” “North America,” “South- or Middle America,” and “Oceania”;

2 Maximum = ‘Above 100 meetings’, *n* = 1 missing;

3 *n* = 22.

**Figure 1.**
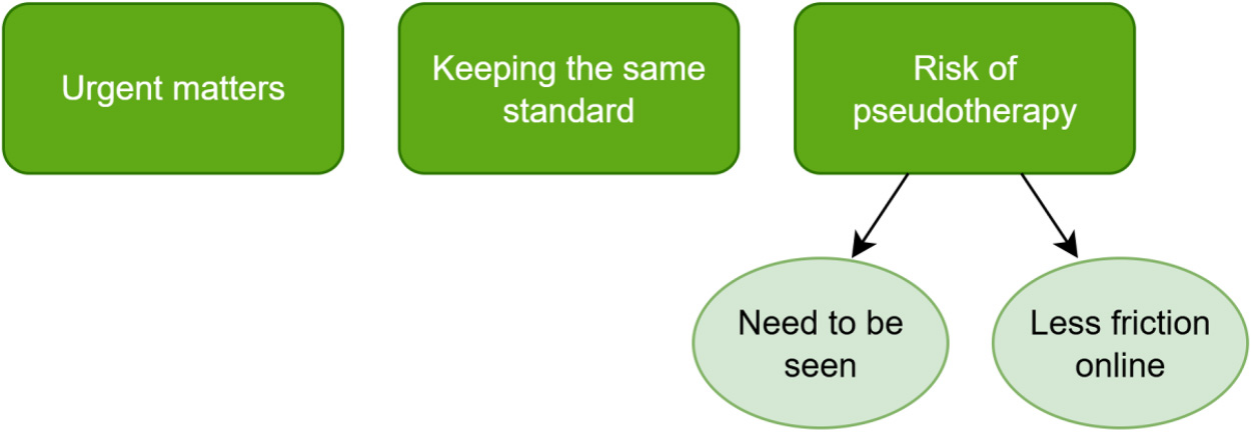
Thematic map.

We identified three themes and two subthemes (Figure 1) that cover different aspects of the research question, with some overlap, based on data from 28 participants. The first theme “Urgent matters” covers participants’ perspectives on how they believe IBT should be structured. The second theme “Keeping the same standard” covers participants’ perspectives on preferred content and communication. As such, the first and second theme relate to how participants believe IBT can be appropriate for their condition and goals. The third theme “Risk of pseudotherapy” covers what the participants perceive to be facilitating or hindering acceptability of IBT for GD, which also informed their ideas for content and structure. This latter theme included two subthemes: “Need to be seen,” which covers participants’ perspectives on preferred content and communication, and “Less friction online,” which covers what the participants perceive as both benefits and drawbacks/challenges of IBT for GD. Table 2 provides a summary of the results and associated implications for internet-based therapy development that are elaborated on in the Discussion.

**Table 2.** Summary of Results and Implications for Internet-Based Therapy Development.

Theme/subtheme	Central organizing concept	Implications for IBT development
Urgent matters	Prioritize gambling related crisis/harm-reduction needs early	Front-load debt/finance guidance, clear crisis options, and early human contact
Keeping the same standard	Online format should not compromise the depth and scope of therapy	Offer techniques addressing both gambling problems and the comorbidity that influences it
Risk of pseudotherapy	Gambling disorder therapy has the risk of falsely appearing effective	Avoid superficial therapy engagement by building accountability, facilitating social support within/outside therapy, and addressing relapse risk
Need to be seen	Securing relational needs is crucial within IBT for gambling problems	Enable social interaction to combat shame, loneliness, gambling concealment, and to increase problem awareness and well-being
Less friction online	Accessibility in IBT is double-edged	Maintain a low barrier for therapy, but prevent avoidance/low adherence by having contact/testimonials from others with lived experience with gambling problems

*Note.* IBT = Internet-based therapy.

### Theme 1: Urgent Matters

Participants’ responses reflected the view that certain needs are more immediate and that the beginning of therapy represents a critical phase for those seeking help for gambling problems. The central organizing concept of this theme is that prioritizations must be made for structuring IBT for GD. Participants appeared to emphasize crisis management issues that the internet-based format was anticipated to handle inadequately. Many participants emphasized the importance of receiving assistance with practical issues, particularly help with personal finances and gambling debt. For example, one participant stated that the following should be prioritized in the beginning of therapy:

Economy, relationships to next of kin, where one can get help with critical/acute aspects related to bills, food, etc. (especially important when one has standard minimum amounts for livings expenses and higher costs in [to living] in the society). (#4, Man, Lifetime gambling problems)

The participants’ statements can be understood to mean that internet-based solutions must be structured to address this through either therapist-assistance or coordination with other services. This was made explicit by several participants. For example, one participant followed up by stating that these immediate concerns need to be addressed for the individual to be able to fully engage in psychological treatment:

Often there are persons who also struggle a lot financially due to debt so also [being able to] receive guidance/help to organize a payment plan with creditors and a livable budget is just as important. This can relieve the consequences associated with the gambling addiction that many gamblers experience which makes it easier to concentrate and put energy towards becoming free from gambling. (#20, Man, Lifetime gambling problems)

A few participants communicated that the need for therapist assistance is greatest in the beginning when comorbid mental health problems and suicidality can be most pressing. When asked about how the therapy should be organized, one participant noted perceived limits to IBT and said:

Nr. 1: Mental health. This is because the person is typically really low. I experienced suicidal thoughts and wanted to get rid of these […] Mental health should be treated in physical [i.e., face-to-face] treatment. If you struggle with other things, in addition to gambling, you must get professional help from a psychologist. (#23, No demographic information)

### Theme 2: Keeping the Same Standard

Participants described a range of topics they believed should be included in IBT. The central organizing concept of this theme is that therapy format should not compromise the depth and scope of therapy, a sentiment that was evident across participants’ statements on therapy content. That is, internet-based GD therapy must offer the same therapeutic standards and core components as traditional face-to-face GD treatment for it to be acceptable.

Participants stated that IBT for GD should address situations/triggers for gambling, activity replacement, gambling-related thoughts and urges, alternative coping strategies, the relationship between emotions and gambling, concerned significant others, and financial difficulties, as well as facilitate self-reflection and problem acknowledgement. Each topic was highlighted by several participants, except for financial difficulties which were mentioned by most participants. Patterns in participants’ responses on financial difficulties are covered in the previous theme.

Most participants suggested multiple topics for therapy inclusion and several participants framed these suggestions as a response to viewing GD as a complex disorder. Responding to the question of whether GD therapy should address topics beyond gambling itself, one participant stated:

Yes, gambling brings a lot of other things with it. This was perhaps what I felt was most unfortunate when I was in face-to-face treatment. Everything had to revolve around the gambling, but that may not solve the core of the problem […]. (#10, Man, Current gambling problems)

This participant went on to suggest depression, anxiety, suicidality, self-worth, and hope as important topics for therapy. Another participant, who similarly suggested multiple content ideas and emphasized the complexity of GD, also tackled the risk of program bloat by recommending a chronological structure that lets users opt in or out of each topic.

### Theme 3: Risk of Pseudotherapy

This theme covers a risk for GD therapy failure that was emphasized by participants and how they believe it should be avoided in IBT. The theme's central organizing concept concerned the risk of GD therapy falsely appearing effective (i.e., being pseudotherapy). Participants’ statements reflected this idea when referring to half-hearted therapy engagement, failures to instill a sense of ownership of the gambling problem, false sense of reassurance among significant others, and superficial therapy content. When describing how to avoid mediocre therapy effects, the participants emphasized the need to accommodate for the long-term nature of GD, the need to address the cause of GD, the importance of social interactions, and the accessibility of therapy. The two latter points are elaborated in two sub-themes below. The current theme covers a specific challenge to therapy acceptability, which distinguishes this theme from “Keeping the same standard” because that theme captures participants’ expectations for therapy content more broadly.

Many participants described GD as a long-term or even chronic condition. GD therapy must address this to avoid relapses and IBT was in this realm seen as more susceptible to failure. The following quote was made in response to the vignette:

Place strong emphasis on the dangers and likelihood of relapse. For Petter to be able to stop gambling, he must make enormous changes in his life – both mentally and physically in terms of interests, time use, openness, and dialogue, etc. These are major processes that takes time and are incredibly difficult to work through. An internet-based course will likely struggle to meet these needs. This must be made clear. The alternative – and what can easily happen – is that the gambler deceives himself by only half-heartedly engaging, continues to deceive others, and the people around him are given false hope, thinking: “Well, now he's in treatment, so everything will be fine.” (#2, Man, Current gambling problems)

Most participants stated that GD therapy must address and uncover the underlying cause(s) of the gambling problem. We identified a pattern across statements that these participants perceived this as a core part of effective GD therapy. Answering the question of what is important to include in IBT of GD, one participant said:

Underlying reasons for gambling? Why did one start gambling? In what situations did one gamble? The reason is that I believe we need to try to understand why and where people gamble to implement the right measures, and to help those with gambling addiction become aware of situations they should avoid. (#17, Man, Lifetime gambling problems)

### Theme 3; Subtheme 1: Need to be Seen

The central organizing concept of this theme is the belief that it is crucial to secure relational needs within IBT for those struggling with gambling. We identified an expectation that the therapy must supply or otherwise enable social interaction to alleviate gambling harms and increase well-being. Several participants conveyed concerns that IBT runs the risk of being impersonal, leading to them feeling lonely, shameful, and less accountable in the recovery process. One participant cautioned that individuals with GD are vulnerable to feeling isolated in general. In response to the question on benefits and drawbacks of a non-guided treatment program:

[A] drawback is that a gambling addict is often isolated and if this [treatment] happens without human contact then that will not help with the isolation. (#27, Woman, lifetime gambling problems)

The importance of social interaction was stated in different ways. Several participants conveyed that it was important to openly share the gambling problem with others. A few clarified that such sharing facilitates relatedness, problem awareness, and combats gambling-related concealment and lying. In response to the vignette:

The biggest challenge that he will meet is to be honest with himself and his problems, in addition to being honest with the therapist. A gambling addict will have problems with seeing that they have a problem and have extremely good excuses to maintain gambling. (#13, Man, lifetime gambling problems)

Several participants expressed concern that IBT would be unable to evoke the same type of emotional response as individual or group face-to-face therapy. Relatedly, several participants believed that an emotionally supportive therapist or others with lived experience with GD could help alleviate negative feelings of loneliness and shame. The therapist can also offset the risk of IBT being experienced as impersonal:

Support must come through familiarity with the patient. Also, having a real person to relate to. The patient has likely spent enough time alone, and as of today, a machine cannot replace human emotions or the way a patient should be understood. (#7, Man, lifetime gambling problems)

People with previous GD may also share their story through video content to obtain a similar effect, as suggested by one participant.

### Theme 3; Subtheme 2: Less Friction Online

Many participants stated that the benefit of IBT for GD is that it is a highly accessible form of therapy. These participants referenced greater geographical coverage of therapy, time flexibility, opportunities to combine therapy with other responsibilities, and aspects related to anonymity. The central organizing concept of this theme is frictionless therapy because this captures both perceived benefits and challenges with IBT. Greater treatment accessibility through internet-therapy was mostly emphasized in responses to the question about perceived benefits and drawbacks of an unguided version of the therapy. However, IBT being a rather anonymous form of therapy, was described as a double-edged sword:

We as gambling addicts tend to prefer quick fixes and have often relied on lies to keep gambling. With anonymous treatment, I worry that they might make hasty decisions that would be harder to make with a physical person. One advantage, though, is that those who are very fearful and ashamed may find it easier to open up when there is no direct contact. I would say this is very individual, but I, who really didn’t want to talk to a person, am very grateful that I did. (#11, Man, lifetime gambling problems)

Several participants communicated similarly that they believed the benefits of IBT, particularly the unguided variant, were limited to the first phase of help-seeking where it can alleviate shame and stigma as treatment barriers. These participants stated that once IBT starts, drawbacks related to the internet format and/or anonymous aspect would emerge. A few participants specified that such therapy would lead to less accountability for own gambling behavior and/or higher risk for relapses. The benefits of social interaction with a therapist or those with lived experience with GD, as detailed in the theme “Need to be seen,” was emphasized by several participants when explaining the drawbacks. This includes less accountability as a drawback, for example:

The downside is that you don’t get any sense of ownership. It feels like you’re talking to a robot. I prefer a face when going to treatment. The upside is that your workplace won’t find out anything. If you want to keep it within your own four walls, you have the opportunity to do so when the treatment is anonymous. (#23, No demographic information)

## Discussion

The aim of the present study was to investigate what individuals with current and life-time gambling problems identify as important elements for IBT for GD to be appropriate for their condition and goals, including what facilitates or hinders such acceptability. One of the salient findings concerned participants’ views on IBT structure, including the risk of lower acceptability by failing to account for GD as a complex condition with goals beyond reducing excessive gambling. The findings within the theme “Urgent matters” indicated that individuals struggling with gambling may approach therapy with a sense of desperation and with the expectation that therapy should address gambling harms in addition to GD itself. Gambling can harm multiple areas of life for the gambler and concerned significant others, including finances, work/studies, physical and mental health, relationships, and law abidingness, as well as the community (Langham et al., 2016; Li et al., 2017; Syvertsen et al., 2023). Previous research indicates that many struggling with gambling wait until their problematic behavior and associated harms become severe before seeking treatment (Loy et al., 2018). Participants in the present study also highlighted suicidality which represents a serious issue for this patient group where the lifetime prevalence of suicidal ideation is estimated to 32% and lifetime suicide attempts to 13% (Kristensen et al., 2024). The findings can be taken to suggest that acceptability can be improved by communicating in recruitment material and within the IBT program that debt, finances, and crisis management for gambling harms will be addressed early in the program to combat hopelessness as a potential barrier for treatment seeking. Participants suggested that addressing such harms could include therapist involvement or being connected to other additional services (e.g., financial advisors, family counselor services, mental health lines).

Participants had varied suggestions for content that could make IBT for GD more acceptable. These suggestions, as described in “Keeping the same standard,” also coincide with what is typically offered in GD therapy and for CBT (Rodda et al., 2018). Participants’ ideas for content may be influenced by their own therapy experience. Of all the participants, only four had experience with IBT, whereas most had experience with face-to-face therapy (20 out of 23 reporting background information). CBT is the most prevalent GD therapy offered in Norway (Torvund et al., 2018). It is possible that the participants suggested content based on what was familiar to their own therapy experience and that treatment-naïve individuals could have provided different suggestions. Regardless, participant statements revealed an overarching expectation that online format should not compromise the quality of therapy. This aligns with findings in previous studies on expected acceptability among the general population and GD individuals who are concerned that IBT may be less professional and effective compared to face-to-face therapy (Hawker et al., 2025a; Lincke et al., 2022).

Overall, participants expressed concern and ambivalence towards IBT for GD. These sentiments were elaborated upon in the theme “Risk of pseudotherapy” and the accompanying sub-themes. Central to these concerns was the absence of social interaction in IBT, whether with a therapist or with peers, which was seen as crucial for reducing isolation and fostering problem understanding, self-efficacy, and motivation. This aligns with previous evidence on the role of social support in GD recovery (Gomes & Pascual-Leone, 2009; Petry & Weiss, 2009) and with findings that self-help group members place high value on interpersonal factors (Syvertsen et al., 2020). The efficacy of IBT for GD is well-supported, although potential users of IBT for GD may need assurances that the program will also address social support in some way. Indeed, IBT with therapist support shows higher treatment effects than non-guided IBT (Sagoe et al., 2021). In addition to therapist involvement in guided formats, the need for personal support might partly be addressed by using audio and video content featuring people with lived experience, as was also suggested by one participant.

Participants described a duality to IBT: ease of access makes it easier to start but difficult to sustain, especially in unguided formats. In other words, noting an IBT aspect that both facilitates and hinders acceptability. Difficulties for IBT in sustaining patient engagement is supported by prior studies showing lower adherence in unguided interventions (Ebert et al., 2018) and limited activity engagement even when guidance is offered (Dowling et al., 2018). Although IBT engagement can be a challenge across different psychological disorders, addictive disorders such as GD feature more avoidance and concealment, which can explain why participants emphasized the importance of keeping patients accountable throughout the IBT process (Potenza et al., 2019). The results from the present study suggest that avoidance and/or low therapy adherence might be alleviated by providing social interaction or testimonials from others with lived experience with GD.

### Conceptualizing Acceptability in Internet-Based and Other Services for Problem Gambling

The present findings can also be interpreted through the Theoretical Framework of Acceptability of Sekhon et al. (2017), helping to specify what acceptability means in the context of IBT for GD. Overall, participants were skeptical about IBT's capacity to support sustained recovery, at the same time as offering concrete suggestions for making IBT more acceptable (barriers and facilitators for perceived effectiveness). Suggestions to include therapist guidance, resources for concerned significant others, and support for comorbid conditions to improve perceived effectiveness mirrors previous research on IBT for GD (Sanchez et al., 2019). Their recommendations regarding structure, prioritization, and which topics IBT should cover suggest that participants largely understood what IBT can offer (intervention coherence). The strong emphasis on front-loading financial guidance, crisis management, and early therapist contact highlights the need to make the initial treatment phase workable in chaotic life circumstances (reducing burden and increasing self-efficacy). Participants also stressed that IBT must be designed to remain effective over time, not merely lower the threshold for entering a superficial program (perceived effectiveness and burden). In addition, descriptions of IBT as being potentially impersonal or conducive to loneliness indicate clear image-related issues for developers to address (affective attitude). Finally, participants not only recognized that IBT may reduce practical costs (e.g., travel time and scheduling barriers), but also noted possible motivational trade-offs because engaging in IBT may require more independent effort (reduced and increased opportunity costs).

Many of these acceptability issues are not exclusive to IBT and the issues are associated with concrete outcomes and current challenges such as help-seeking and drop-out for GD. Help-seeking for gambling problems is estimated to be as low as 20%, which includes formal treatment and other services (e.g., contact with social workers, self-help groups, physicians) (Bijker et al., 2022). Crises, financial difficulties, problem severity, and emotional difficulties emerge as the important motives for seeking help for people struggling with gambling (Loy et al., 2018). This underscores the importance of the current participants’ calls for greater prioritization of crisis management and financial guidance in IBT for GD. Help-seeking is a key challenge that digital interventions for mental health, including IBT, have been promoted to address, with interventions promoting active participation and personal involvement being most promising (Evans-Lacko et al., 2022). Drop-out rates are estimated to be as high as 39% for face-to-face GD treatment (Pfund et al., 2021). A recent review found that practical issues (e.g., scheduling conflict, work and family commitments) were the most common reason for dropout from GD therapy, with other reasons including comorbidity and lack of motivation or progress (Hawker et al., 2025b). IBT could be better positioned to alleviate practical issues driving dropout, but other challenges such as digital literacy could explain why IBT and face-to-face mental health treatment even out to similar levels of adherence and drop-out rates (Nilsson et al., 2023; Van Ballegooijen et al., 2014).

IBT has been promoted partly because it can be delivered cheaper than face-to-face therapy, especially when human contact is reduced or removed (McDonald et al., 2020). The appeal of such low-cost alternatives may grow when public budgets tighten, but participants’ statements caution against removing features such as therapist guidance and early crisis and financial support. Cost-effectiveness must be weighed against user preference and equity (Buntrock, 2024). Omitting these features risks an intervention that is less acceptable to users, overlooks more disadvantaged groups with the most widespread gambling harms, and may prove less cost-effective for welfare systems long term if recovery is not sustained and the socioeconomic costs of gambling problems are prolonged (Kristensen et al., 2022).

### Strengths and Limitations

The present study used a qualitative survey, which can reach a wider audience that is more geographically dispersed compared to the audience typically reached with interviews (Braun et al., 2021; Thomas et al., 2024). This is a strength when targeting limited populations that are spread across large areas geographically, as is the case for those struggling with gambling in Norway. Qualitative surveys rely on written responses and offer convenience and complete anonymity, making it easier to participate at a time and place seen fit and share opinions on sensitive topics. These characteristics alongside open-ended questions can make it easier for participants to also express disagreement with the (perceived) researcher position (Thomas et al., 2024). This was relevant in the present study because ambivalent and/or negative perceptions about IBT were important to capture. Qualitative research favors long response answers that can supply rich data, although qualitative surveys have been criticized by some as being susceptible to poorer quality data due to lack of follow-up and probing questions. Although there exist rebuttals to this criticism (Braun et al., 2021), we also opted to use the vignette method as an elicitation tool for longer responses/richer data and to increase participants’ consideration of typical GD characteristics such as motivational ambivalence, relapse, loss of control, and pre-occupation with gambling (Barter & Renold, 1999; Thomas et al., 2024). We could not assess how successful the vignette was in promoting these goals compared to having no vignette, although the vignette was rated highly realistic by the participants. Finally, qualitative surveys rely on written responses meaning individuals with limited literacy skills may experience difficulties expressing themselves as rich and spontaneous as they would in oral interviews or choose to opt out of the survey as a result (Braun et al., 2021). The sample should be considered when interpreting the present findings. The present study differentiated people currently or previously struggling with gambling more than 12 months ago, although greater nuance could have been achieved by assessing the length of time since struggling with gambling previously. Overall, the study sample can be considered fit to investigate expected acceptability of IBT understood as prospective perceptions among potential users, with only a minority having any experience with IBT (four out of 23 providing background information). The present study also consisted of individuals who attend self-help groups, which may make them more inclined to value factors that are emphasized for recovery within self-help groups (e.g., such as openness and sharing with others) above factors more characteristic for individual psychotherapy (Humphreys & Rappaport, 1994; Schuler et al., 2016; Syvertsen et al., 2020). This could then result in more skepticism towards individual IBT as it constitutes (guided) self-help. This is not necessarily a study weakness but speaks to a unique perspective among self-help group participants. Future studies should complement these perspectives with that of individuals with lived experience on GD, but with different or no help-seeking experience.

### Future Research and Conclusions

The present study found that the participants preferred content that matches what is typically offered in CBT for GD; however, their ideas for therapy structure called for a greater prioritization of crisis management and gambling harm alleviation early in therapy. The participants anticipated that IBT for GD can ease access to therapy, but that this benefit is offset by a lower ability to sustain therapy engagement. The challenge of IBT engagement was understood as being influenced by GD characteristics. Patients were assumed to engage in avoidance because of motivational ambivalence and social contact with a therapist or those with lived experience was seen as necessary to combat this. Following this, the findings suggest that conventional measures of IBT acceptance may overlook GD-specific concerns.

Future qualitative research may investigate how individuals struggling with gambling construct meaning around the identified concerns; for example by conducting interview studies where one can ask follow-up and probing questions on the topics. Future quantitative research on IBT acceptance among individuals struggling with gambling could supplement validated instruments such as the Attitudes towards Psychological Interventions Questionnaire (Schröder et al., 2015) with additional questions on IBT's ability to accommodate loneliness, isolation, and avoidance. The present study and a previous study also suggests that comorbidity and concerned significant others are important topics for individuals with gambling problems when evaluating IBT acceptability (Sanchez et al., 2019). Finally, studies may also investigate the effect of marketing campaigns designed to increase expected acceptability by targeting the concerns identified here. Previous campaigns using video or text have shown small but positive effects on IBT acceptance (Apolinário-Hagen et al., 2018; Casey et al., 2013). Overall, the present findings have implications for how IBT for GD is designed and communicated to potential users to increase acceptability and engagement. Stakeholders should emphasize crisis management, the program's long-term recovery benefits, built-in social support, and features that help users sustain engagement over time

## Supplemental Material

sj-docx-1-nad-10.1177_14550725261465112 - Supplemental material for Acceptability of Internet-Based Therapy: Considerations Expressed by Individuals Struggling with Gambling Supplemental material, sj-docx-1-nad-10.1177_14550725261465112 for Acceptability of Internet-Based Therapy: Considerations Expressed by Individuals Struggling with Gambling by André Syvertsen, Bjørn Solberg-Hansen Fondenes and Ståle Pallesen in Nordic Studies on Alcohol and Drugs
