# Supplementary material for: Acceptability of Internet-Based Therapy: Considerations Expressed by Individuals Struggling with Gambling
Source: Nordisk Alkohol Nark. 2026 Aug 10:14550725261465112. Online ahead of print. doi: 10.1177/14550725261465112 (PMC13457415; doi:10.1177/14550725261465112)
Supplement: Supplementary file 1 [file sj-docx-1-nad-10.1177_14550725261465112.docx]

Supplemental material – English translation of qualitative survey questions and vignette

**Views on Internet-Based Treatment for Gambling Problems**

**Part 1 – Questions about internet-based treatment for gambling problems in general:**
“Internet-based treatment for gambling problems refers to treatment that takes place online via PC, mobile phone, or tablet. The treatment may involve therapist contact or not, where contact can include messaging, video, and/or phone calls. In such treatment, one typically works with written material and exercises based on scientifically supported therapies (for example, cognitive behavioral therapy).”

1. If an internet-based treatment program for gambling problems were to be developed:
   What kind of topics do you think should be included? Please explain why you think they are important.
2. What kind of support should a therapist provide in such a program? Please explain why you think it is important.
3. We are considering developing a version of the treatment where those receiving it are not offered contact with a therapist, but where everything takes place anonymously online. What do you think could be the advantages and disadvantages of this?
4. How should the content of the program be organized? Please explain why you think it is important.
5. Should internet-based treatment help the person with something beyond reducing or stopping gambling? If so, what and why?

**Part 2 – Vignette**
In the next questions, we ask you to consider the following situation:

Petter developed an early interest in gambling. He especially remembers a time when he was with his father on the ferry to Denmark and was allowed to try the slot machines. As a teenager, Petter spent his pocket money on slot machines in stores, but did not see it as problematic. He just had to wait until the following week for more money. When the slot machines disappeared from the stores, his interest in gambling suddenly declined as well.

Several years later, Petter came across an advertisement for online slot machines and decided to try them. Gradually, his interest in gambling blossomed again, at the same time as he moved in with his partner and got a steady job. Over time, Petter began spending his entire paycheck on online slots, and his partner noticed that he was very reluctant to spend money on vacations, movie tickets, and groceries. Petter insisted on handling the household bills, something he convinced his partner to let him do. One day, however, his partner came across the letters he had hidden away. All the debt collection notices were revealed.

Petter opened up to his partner about his gambling, and they agreed that he should seek help to regain control. Petter was somewhat skeptical about whether he really needed help, but agreed to participate in treatment for his partner’s sake, if nothing else.

Petter participates in internet-based treatment. In the treatment, he reads and practices specific parts, like chapters. Petter receives information but is also asked to reflect on questions such as: What situations make me vulnerable to gambling? What gambling-related thoughts feel especially tempting to me? What feelings have I tried to create or avoid through gambling? He also receives concrete strategies and techniques to manage gambling urges.

**Continued questions:**

6. What kinds of challenges do you think Petter might face during such a program?
7. How could such challenges be addressed in the treatment program?
8. Is there anything else you would like to share regarding internet-based treatment for gambling problems?
